# Diagnostic characteristics of refractometry cut‐off points for the estimation of immunoglobulin G concentration in mare colostrum

**DOI:** 10.1111/evj.13568

**Published:** 2022-03-08

**Authors:** Elisa Rampacci, Karen Mazzola, Francesca Beccati, Fabrizio Passamonti

**Affiliations:** ^1^ Department of Veterinary Medicine University of Perugia Perugia Italy

**Keywords:** failure of passive transfer, horse, refractometry, ROC curve, sensitivity, specificity

## Abstract

**Background:**

Feeding foals with poor quality colostrum predisposes them to failure of passive transfer (FPT). FPT is a major risk factor for neonatal infections.

**Objectives:**

To assess the optimal cut‐offs for the optical (OR) and digital (DR) refractometer and determine their accuracy for poor quality colostrum diagnosis.

**Study design:**

A diagnostic validation study.

**Methods:**

Eighty‐one colostrum samples and sera were collected from broodmares and their neonatal foals, respectively. Colostral and serum IgG concentrations were measured by radial immunodiffusion (RID), DR and OR. Correlation coefficients were calculated. ROC curves were generated to identify optimal cut‐offs for the refractometers and their diagnostic characteristics were evaluated.

**Results:**

The optimal cut‐offs for DR and OR were ≤23.75% and 23.9%, respectively. The sensitivity and specificity of the DR were 93.3% (95% CI: 66.0‐99.7) and 87.9% (95% CI: 77.0‐94.3) to detect colostral IgG <60 g/L, respectively. The sensitivity and specificity of the OR were 93.3% (95% CI: 66.0‐99.7) and 81.8% (95% CI: 70.0‐89.9), respectively. DR and OR had negative predictive values of 98.3% (95% CI: 89.7‐99.9) and 98.2% (95% CI: 89.0‐99.9), respectively, whilst positive predictive values were lower. No maternal variable, including breed, significantly influenced colostral IgG concentrations. Fifteen out of 81 colostrum samples had IgG <60 g/L. FPT and PFPT were diagnosed in 4/81 and 10/81 foals, respectively. Nine out of 14 animals with FPT/PFPT suckled colostrum with IgG <60 g/L. A moderate correlation (*r*_s_ 0.542; *P* = .01) was observed between IgG concentrations measured by RID in sera and colostrum.

**Main limitations:**

A smaller number of samples than the size requirement based on a priori estimate of specificity and the low prevalence of poor quality colostrum.

**Conclusions:**

The method has the potential to reliably differentiate between good and poor quality colostrum. Assessing colostrum quality by refractometry may be an indicator of passive transfer of immunity.

## Résumé

**Introduction/Contexte:**

Nourrir les poulains avec du colostrum de mauvaise qualité prédispose à l’échec du transfert d’immunité passive (FPT). FPT constitue un risque majeur pour les infections néonatales.

**Objectifs:**

Évaluer les valeurs limites optimales au réfractomètre optique (RO) et digital (RD) et déterminer leur précision pour le diagnostic du colostrum de pauvre qualité.

**Type d’étude:**

Étude de validation diagnostique.

**Méthodes:**

Quatre‐vingt‐un colostrums et sérums ont été recueillis à partir de juments de reproduction et de leur poulains nouveaux‐nés respectivement. Les concentrations d’IgG dans le sérum et le colostrum ont été mesurées par immunodiffusion radiale (IDR), RO et RD. Les coefficients de corrélation ont été calculés. Des graphes d’air sous la courbe (ASC) ont été générés afin d’identifier les valeurs limites optimales aux différents réfractomètres et leurs caractéristiques diagnostiques ont été évaluées.

**Résultats:**

Les valeurs limites optimales pour les RD et RO étaient ≤23.75% et 23.9% respectivement. La sensibilité et la spécificité du RD étaient 93.3% (95% IC: 66.0–99.7) et 87.9% (95% IC: 77.0–94.3) pour la détection des IgG colostraux <6000 mg/dl, respectivement. La sensibilité et spécificité du RO étaient de 93.3% (95% IC: 66.0–99.7) et 81.8% (95% IC: 70.0–89.9), respectivement. Les RD et RO avaient une valeur prédictive négative de 98.3% (95% IC: 89.7–99.9) et 98.2% (95% IC: 89.0–99.9) respectivement, alors que les valeurs prédictives positives étaient plus basses. Aucune variable maternelle, incluant la race, n’a influencé significativement les concentrations colostrales en IgG. Quinze des 81 échantillons colostraux avaient une valeur d’IgG <6000 mg/dl. FPT et PFPT ont été diagnostiqué chez 4/81 et 10/81 poulains respectivement. Neuf des 14 animaux avec FPT/PFPT ont reçu du colostrum ayant des valeurs d’IgG <6000 mg/dl. Une corrélation modérée (rs 0.542; p= 0.01) a été observée entre les concentrations d’IgG mesurées par IDR dans le sérum et le colostrum.

**Limites principales:**

Le nombre d’échantillon est inférieur à celui recommandé basé sur unestimé *a priori* de la spécificité et considérant la faible prévalence de colostrum de pauvre qualité.

**Conclusions:**

La méthodologie utilisée pourrait différencier de façon fiable les colostrums de pauvre et bonne qualité. L’évaluation de la qualité du colostrum par réfractométrie pourrait représenter un indice du transfert d’immunité passive.

## 1 INTRODUCTION

Neonatal foals have negligible concentrations of serum immunoglobulins (Ig) at birth due to the epitheliochorial structure of the equine placenta.^1^, ^2^ As a result, they are considered agammaglobulinemic. Passive immunity is acquired by the foal from the ingestion of colostrum, which contains a mean of 70 g/L IgG at foaling, falling rapidly after 2‐3 h to below 5 g/L at 24 h.^3^ Foals required approximately two weeks to develop a primary immune response.^4^ In this interval, passive immunity is essential for the protection of the animal against environmental opportunistic pathogens. Failure of the foal to ingest or absorb a sufficient amount of colostral IgG results in complete failure of passive transfer (FTP, serum IgG <4 g/L) or partial failure of passive transfer (PFPT, serum IgG between 4‐8 g/L).^5^, ^6^ This immunodeficiency disorder is a major risk factor for the development of neonatal infections.^7^ Individual and breeding management variables have been controversially associated with FPT. Among these, the content of IgG in colostrum may be used to predict FPT being correlated to the serum IgG concentration in foals at 24 h after birth.^8^, ^9^, ^10^ Cash used an IgG concentration cut‐off of >50 g/L to indicate good quality colostrum,^11^ whilst Chavatte used >60 g/L.^12^ Assessing colostrum quality at birth may be useful to identify foals at high‐risk of developing FPT and thus to supplement with an artificial administration of fresh or frozen‐stored good quality colostrum before “gut closure.” Semi‐quantitative optical (OR) and digital refractometers (DR), which measure the concentration of dissolved solids in a solution on a percentage scale, provided an assessment of colostrum quality that was shown to correlate to colostral IgG concentrations measured by radial immunodiffusion (RID) as a gold standard method.^11^, ^12^ Therefore, refractometers are used as cheap and rapid tools to measure colostral IgG concentration, and they exhibited utility as screening tests for assessing FPT in neonatal foals.^13^ There are few studies that assessed the diagnostic potential of OR and DR for determining equine colostrum quality.^11^, ^12^ On this basis, the current study aimed to investigate the optimal cut‐off points for OR and DR in receiver operating characteristic (ROC) curves and to determine their accuracy for poor quality colostrum diagnosis. Colostral IgG concentrations measured by RID and the values read by OR and DR were analysed by correlation test, which also measured the relationship between colostrum and serum IgG concentrations for prediction of FPT development in foals.

## 2 MATERIALS AND METHODS

### 2.1 Animals

**FIGURE 1:**
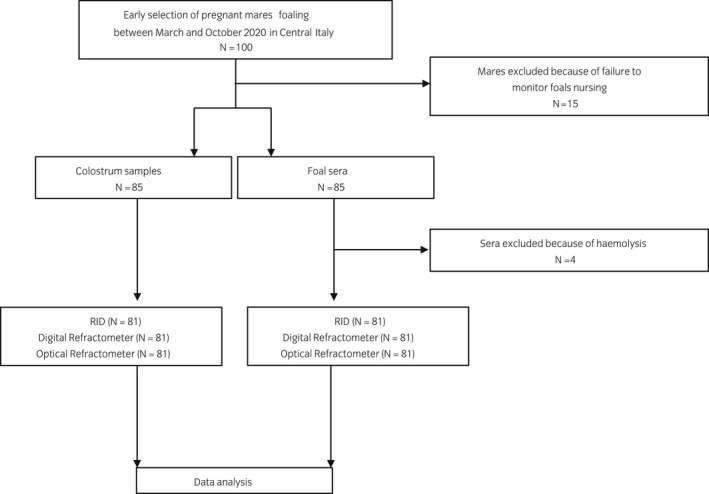
Flow chart illustrating the study population. Colostrum and serum samples were collected from mares and their foals, respectively, and examined by the reference radial immunodiffusion (RID) assay, digital and optical refractometers

The minimum sample size was calculated by the formula for diagnostic test validation using a reference standard,^14^ estimating a method sensitivity of 90%–93% a priori and margin of error of 0.07 at 95% confidence interval. A sample size of ≥71 mares was established as adequate for this study. A total of 100 pregnant mares were selected. Fifteen out of 100 animals were excluded because of failure of monitoring of foals nursing. Additionally, a further four mares were excluded because of sampling error (Figure 1). Eighty‐one broodmares were enrolled in the study: 11 Thoroughbreds (TB), 31 Arabians (Arab) and 39 Standardbreds (STB) were selected as recipients. The broodmares foaled between March and October 2020 in four breeding farms in Central Italy. Age, breed, parity and information about foaling were recorded for each animal. During the last month of gestation, mares had access to pastures during the day and were moved to foaling boxes overnight; they were fed with hay and concentrate two to three times a day, depending on farm management. All broodmares were resident at the respective breeding farm or were transported to the farm at least 4 weeks before the expected parturition date. Colostrum was collected from each mare before allowing the foal to nurse.

Blood samples were collected from the foals at 24 h after birth for routine clinical monitoring of antibody levels. An aliquot of the collected blood was retained for research purposes based on the willingness of the owner to participate in the study, allowed to clot and centrifuged to collect the serum. Samples were refrigerated at 4°C at the farm and transported to the laboratory to be stored at −20°C until analysis. A single sample was collected from each animal.

### 2.2 Colostrum and serum assessment by refractometers

Each sample was analysed at room temperature with a handheld OR (ATC, scale 0%–32%) and with a DR (MISCO Palm Abbe no.PA201, scale 0%–56%). Before each evaluation, both refractometers were calibrated with water and wiped clean after. The operator measured colostrum and serum samples by the OR first to avoid investigator bias. For the OR, one to two drops were placed on the prism and covered with the glass lid. The resulting value was read at the line between light and dark areas through the viewfinder whilst in front of a light source. For the DR, approximately 300 µL of colostrum and serum was used and the score was determined by transmitting visible light through the sample in the prism and recording the index of refraction as refractometry percentage on a digital scale.

### 2.3 Radial immunodiffusion assay

Commercial single RID assay was performed to measure the IgG concentration in colostrum and sera according to the manufacturer's instructions (Horse IgG IDRing^®^ Test, IDBiotech, France).

Briefly, after diluting colostrum 1:600 and sera 1:150 in phosphate‐buffered saline (PBS), 15 µL of each sample and the assay standards at 200, 100, 50, 25 μg/mL of equine IgG were dispensed in RID plate wells then incubated in a humid box at 37°C for 22‐24 h. At the end of the incubation period, the plates were bathed with a 2% acetic acid solution for 1 min to block the diffusion and then rinsed twice with distilled water. Plates were filled again with 5 mL of distilled water and incubated at room temperature for 10‐15 min and then emptied. The diameters of the precipitates were measured with a handheld calliper and recorded. The RID operator was blinded to the results of OR and DR. The IgG concentration of each sample was extrapolated from a standard curve constructed by plotting the diameter means of the assay standards analysed in triplicate versus the corresponding IgG concentration.

### 2.4 Data analysis

Colostrum samples were categorised based on the IgG content measured by RID, establishing an IgG concentration <50 g/L^11^ or <60 g/L^12^ as indicative of poor colostrum quality. These criteria were used to evaluate the diagnostic performances of DR and OR. FPT in foals was diagnosed when serum IgG were <4 g/L, PFPT if between 4 and 8 g/L, adequate IgG transfer if >8 g/L.^5^, ^6^

Statistical tests were applied by JASP (version 0.8.6), SPSS (version 17.0) and MedCalc (Version 20.019) software. Power calculation was performed by the formula for dichotomous tests,^15^ using the sample size (N = 81) in the denominator and estimating a method sensitivity of 93%. Data normality was assessed by the Shapiro‐Wilk test. After running descriptive statistics for frequency observations, ANOVA with post hoc Tukey and Bonferroni and nonparametric Mann Whitney and Wilcoxon tests were used to verify if IgG concentrations in colostrum samples varied depending on breed, age, parity and type of parturition (eutocic or dystocic), as appropriate.

Spearman correlation coefficients (*r*_s_) were calculated to evaluate correlations between colostral IgG concentrations measured by RID and the values read by OR and DR. Correlation tests were also applied to evaluate differences between colostral and serum IgG concentrations, DR and OR scores. The correlation was interpreted as previously reported^16^, ^17^; *r*_s_ 0.00‐0.30 negligible, 0.30‐0.50 low, 0.50‐0.70 moderate, 070‐0.90 high and 0.90‐1.00 very high.

ROC curves were generated to identify the optimal cut‐off values of poor colostrum quality for the OR and DR. The area under the curve (AUC) was calculated as a measure of good separability, which was defined as the ability of the test to classify colostrum quality, as well as the cut‐off corresponding to the point with the lowest distance to the upper‐left corner of the ROC curve and the highest Youden index. Diagnostic test characteristics of DR and OR at the cut‐offs found were evaluated based on reporting sensitivity, specificity, positive predictive value (PPV), negative predictive value (NPV), positive and negative likelihood ratios with a 95% confidence interval (CI). PPV and NPV were plotted against the prevalence of colostrum samples with IgG concentrations <50 g/L and <60 g/L using MedCalc. Results of statistical analyses with *P* < .05 were considered statistically significant.

## 3 RESULTS

### 3.1 Study population

One hundred pregnant mares were selected for this study. After excluding 19 mares because of failure of monitoring of foals nursing or sampling error, a total of 162 samples were included in this study: 81 colostrum samples from mares and 81 sera from their newborn foals (Figure 1), with a calculated test power of 89.3%.

IgG concentrations measured by RID in colostrum and sera and values read by the OR and DR are shown in Table S1. Whilst data for IgG concentrations measured by OR and DR were not normally distributed (*P* ≤ .004), the values of IgG obtained by RID were normally distributed (*P* = .449).

The average IgG concentration measured by RID in mare colostrum was 95.91 ± 45 g/L with variability among breeds that was not statistically significant; 93.53 ± 32.43 g/L for Arab, 73.14 ± 37.11 g/L for TB and 104.24 ± 53.46 g/L for STB. Maternal age, parity and dystocia neither influenced colostrum quality in the statistical analyses (Table 1). IgG concentrations <50 g/L were measured by RID in 9/81 colostrum samples. Fifteen of 81 colostrum samples had IgG <60 g/L.

**TABLE 1:** Variation of the colostrum quality measured by radial immunodiffusion associated with maternal factors

Variable	Number of samples	IgG < 50 g/L(n)	IgG < 60 g/L (n)
Breed			
Arabian	31	3	3
Thoroughbred	11	3	4
Standardbreds	39	3	8
Age			
<8	36	5	8
8‐14	36	3	5
>14	9	1	2
Parity			
Primiparous	19	2	3
Multiparous	62	7	12
Parturition			
Eutocic	78	8	14
Dystocic	3	1	1

The average serum IgG concentration measured by RID was 15.49 ± 7.2 g/L; 14.53 ± 7.6 g/L for Arab, 12.64 ± 7.7 g/L for TB and 17.07 ± 6.6 g/L for STB.

FPT and PFPT were diagnosed in 4/81 and 10/81 foals, respectively. Three of the four foals with FPT suckled colostrum of poor quality (<50 g/L). Colostrum with IgG concentrations <50 g/L was ingested by 4/10 foals with PFPT, whilst 6/10 animals with PFPT suckled colostrum with IgG <60 g/L. One out of the four foals with FPT and 4/10 foals with PFPT suckled colostrum of adequate quality.

### 3.2 Correlation coefficients

**FIGURE 2:**
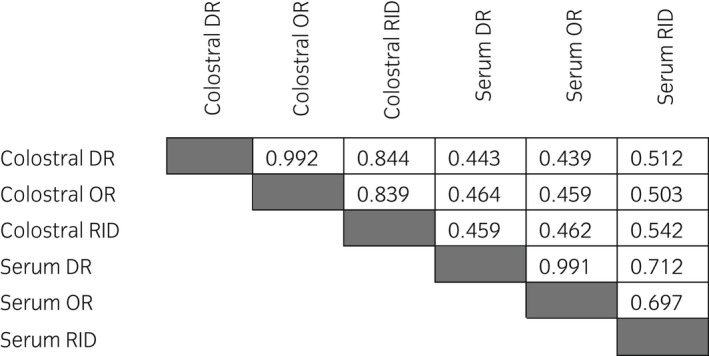
Spearman correlation coefficients between IgG concentrations in colostrum and sera measured by radial immunodiffusion (RID), optical (OR) and digital (DR) refractometers. All correlations were significant at *P* =.01

Spearman coefficients were determined using correlations plots of 81 colostrum samples and 81 sera between (a) DR and OR scores; (b) results of RID assay and DR; (c) results of RID assay and OR score. The results are shown in Figure 2. Colostral IgG concentrations measured both by the DR and OR showed a high correlation with RID values (*r*_s_ 0.844 and 0.839, respectively; *P* = .01). The correlation between the scores provided by the DR and OR for colostrum (*r*_s_ 0.992; *P* = .01) and serum (*r*_s_ 0.991; *P* = .01) was very high. A high positive correlation (*r*_s_ 0.712; *P* = .01) was found between serum RID IgG concentrations and serum DR scores, whilst *r*_s_ of 0.697 suggested a moderate/high correlation between RID IgG concentrations and those measured by the OR. A moderate correlation (*r*_s_ 0.542; *P* = .01) was observed between IgG concentrations measured by RID in serum and colostrum.

### 3.3 Optimal cut‐off points and diagnostic test characteristics

#### 3.3.1 Digital refractometer

**FIGURE 3:**
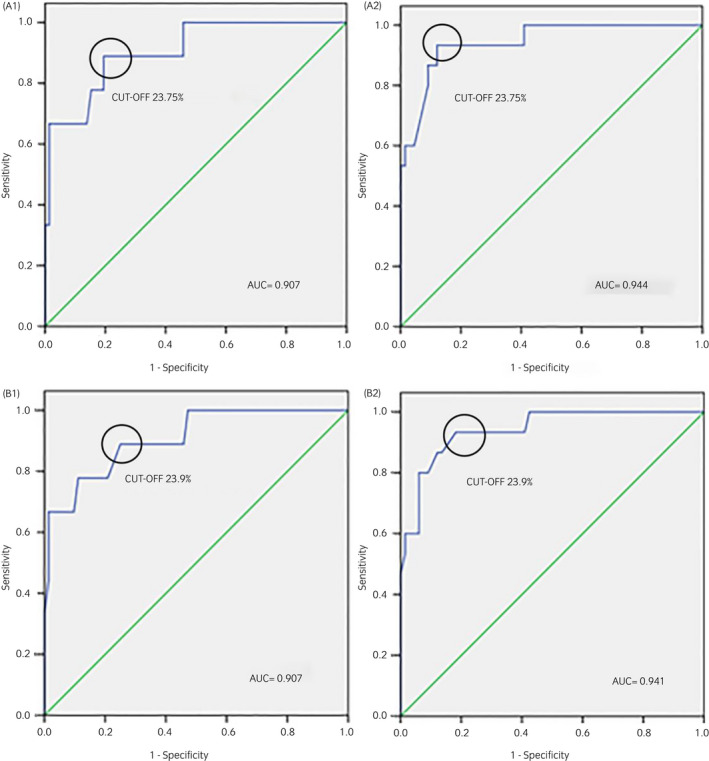
Receiver operating characteristic curves for the digital (A) and optical (B) refractometer to detect colostral IgG concentrations of <50 (A1, B1) and 60 (A2, B2) g/L measured by radial immunodiffusion

**FIGURE 4:**
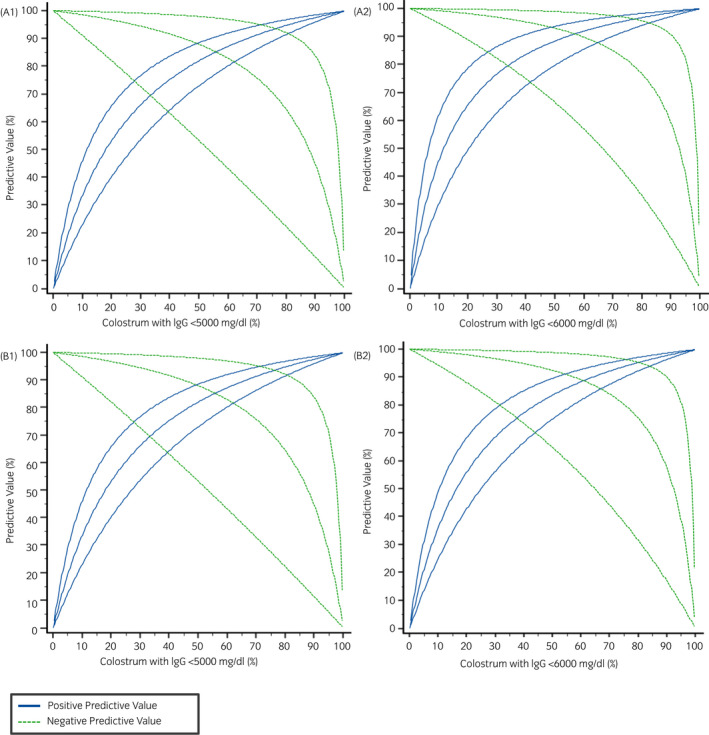
Graph depicting the effect of the prevalence of colostrum samples with IgG concentrations of <50 (A1, B1) and 60 (A2, B2) g/L on positive and negative predictive values calculated for the digital (A) and optical (B) refractometer and their 95% confidence intervals (thin lines). IgG concentrations <50 g/L were measured by RID in 9/81 (11.1%) colostrum samples. Fifteen out of 81 colostrum had IgG <60 g/L (18.5%)

The optimal cut‐off generated by ROC analysis was ≤23.75% to detect concentrations of colostral IgG both of <50 and <60 g/L by DR, with Youden indexes of 0.7 and 0.81, respectively. ROC curves are displayed in Figure 3 (A1, A2). Curve AUC of ≥0.907 indicated a good test separability. DR had a sensitivity, specificity, PPV and NPV of 93.3%, 87.9%, 63.6% and 98.3%, respectively, at the cut‐off of ≤23.75% for the detection of colostral IgG <60 g/L, whereas the diagnostic accuracy of the DR for the detection of colostral IgG <50 g/L using the same cut‐off was lower (Table 2). Using the DR cut‐off of ≤23.75%, false‐positive results were between 8‐14 out of 81 colostrum samples (9.9%–17.2%) depending on whether IgG concentrations <60 or 50 g/L were considered indicative of poor‐quality colostrum, respectively. Only one colostrum sample out of 81 (1.2%) was a false negative. The effect of the prevalence of colostrum of poor quality on NPV and PPV is shown in Figure 4 (A1, A2).

**TABLE 2:** Diagnostic test characteristics of the optical (OR) and digital (DR) refractometer for the detection of colostral IgG concentrations of <50 g/L and 60 g/L at the optimal cut‐off points

	Sensitivity (95%CI)	Specificity (95%CI)	PPV (95%CI)	NPV (95%CI)	Likelihood Ratios (95%CI)		Youden index
					Positive	Negative	
Cut‐off ≤23.75% (DR)							
RID IgG <50 g/L	88.9 (50.7‐99.4)	80.6 (69.2‐88.6)	36.4 (18.0‐59.2)	98.3 (89.7‐99.9)	4.6 (2.7‐7.7)	0.14 (0.0‐0.9)	0.7
RID IgG <60 g/L	93.3 (66.0‐99.7)	87.9 (77.0‐94.3)	63.6 (40.8‐82.0)	98.3 (89.7‐99.9)	7.7 (4.0‐15.0)	0.08 (0.0‐0.5)	0.81
Cut–off ≤23.90% (OR)							
RID IgG <50 g/L	88.9 (50.7‐99.4)	75.0 (63.2 ‐84.1)	30.8 (15.1‐51.9)	98.2 (89.0‐99.9)	3.6 (2.2‐5.6)	0.15 (0.02‐0.9)	0.64
RID IgG <60 g/L	93.3 (66.0‐99.7)	81.8 (70.0‐89.9)	53.8 (33.7‐72.9)	98.2 (89.0‐99.9)	5.1 (3.0‐8.7)	0.08 (0.01‐0.5)	0.75

Abbreviations: 95%CI, 95% confidence interval; NPV, negative predictive value; PPV, positive predictive value.

#### 3.3.2 Optical refractometer

The OR performed comparably to the digital one. The AUC of the ROC curves were ≥0.907, supporting the goodness of the statistical model. ROC analysis identified an optimal cut‐off of ≤23.9% for the detection of IgG concentrations in colostrum both of <50 g/L and <60 g/L, with Youden indexes of 0.64 and 0.75, respectively. Similarly to the DR, sensitivity, specificity, PPV and NPV of the OR for the detection of colostral IgG <60 g/L were 93.3%, 81.8%, 53.8% and 98.2%, respectively, whilst the diagnostic accuracy using the cut‐off of ≤23.9% for the detection of colostral IgG <50 g/L was lower (Table 2). Using the optimal cut‐off of ≤23.9%, OR generated false‐positive results between 12 and 18 out of 81 colostrum samples (13.8%–22.2%) depending on whether IgG concentrations <60 g/L or 50 g/L were considered indicative of poor‐quality colostrum, respectively. Only one colostrum sample out of 81 (1.2%) was misdiagnosed as a false negative. Figure 4 (B1, B2) illustrates the relationship between the prevalence of colostrum samples with IgG concentrations <50 or 60 g/L and predictive values at 95% CIs.

## 4 DISCUSSION

In addition to delay in suckling or failure of intestinal absorption of IgG, feeding foals with low‐quality colostrum predisposes them to neonatal infections.^18^, ^19^ Monitoring colostrum quality and proper intake is pivotal to protect foals in the early days of life. Several studies have shown the efficacy of refractometers for the easy stall‐side assessment of colostrum quality in different animal species. In cattle, it was hypothesised that some variables, particularly breed, may influence IgG concentrations in colostrum and thus the definition of the optimal cut‐off point for low‐quality colostrum diagnosis.^20^ In the current equine study, no maternal variable was found to influence colostral IgG concentrations significantly. Therefore, the optimal cut‐off points for the detection of poor‐quality colostrum may apply to horses regardless of individual factors.

Information on the interpretation of OR and DR scores for equine colostrum have been available in the literature since the late 90s. Chavatte found that OR values of ≥23% were indicative of a good quality of colostrum (IgG >60 g/L) collected from mixed Warmblood breeds and trait Breton mares.^12^ For DR, interpretative ranges were proposed, with percentage values between 20% and 30% suggesting an adequate quality (colostral IgG between 50‐80 g/L).^11^ With ROC analysis, we calculated cut‐off points of ≤23.75% and ≤23.90% for poor colostral quality diagnosis by DR and OR, respectively. Our results are thus consistent with previous guidelines. Our results also suggested that individual factors were associated with minimal variations of IgG concentrations in colostrum and, consequently, of diagnostic refractometry cut‐offs for horses and that the calculated cut‐offs seem to detect IgG concentrations in colostrum of <60 g/L more accurately than <50 g/L.

Although it does not replace measurement of neonatal serum IgG concentrations, accurate colostrum assessment can highlight foals that have suckled adequate colostrum and if poor‐quality colostrum is detected, colostrum supplement may be added to avoid FPT. PPVs for the prediction of poor colostral quality with the proposed diagnostic methods were between 30.8% and 63.6%, resulting in 36.4%–69.2% of false‐positive samples depending on the refractometer used. On the contrary, the high NPVs for the prediction of poor colostral quality obtained for both refractometers using the established cut‐offs can reduce false‐negative results. In our study, only one of the 81 (1.2%) colostrum samples was misdiagnosed as a false negative. This is important because if colostrum is estimated to be of poor quality, alternatives such as colostrum from another mare or frozen colostrum may be administered promptly before “gut closure.”

However, our results should be treated with caution. IgG concentrations <50 g/L were measured in 9/81 colostral samples, whilst 15/81 samples had IgG <60 g/L, indicating a low prevalence of colostrum of poor quality in our population. This affected the width of the margins of error obtained at 95% CI, particularly for predictive values (Table 2), inferring low precision of the estimates. As a result, generalisability is limited and requires data on larger populations. Incorporating an estimated prevalence in the target population in sample size calculation may provide more precise estimates of the diagnostic test characteristics for the refractometers with little margin of error.^21^ An additional limitation of the study is the smaller number of samples based on a priori estimate of specificity of about 80%–85%. A sample size of 81 mares was established as adequate for this study for an a priori estimate of sensitivity. Sensitivity is commonly used for sample size calculations.^14^ However, some authors suggest separate sample size calculations for sensitivity and specificity.^22^

The OR performed similarly to the digital tool (*r*_s_ 0.992 *P* < .01), even though the DR had better diagnostic performance (Table 2). The RID method is the gold standard for the measurement of the IgG concentration in colostrum and serum. As a result, finding that OR and DR indexes for colostrum and serum are highly correlated to RID results (Figure 2) supports the use of the two refractometers for colostral assessment^12^ and as rapid and inexpensive screening tests for FTP in neonatal foals.^13^

Evaluation of colostrum quality by a refractometer could be potentially used to predict FPT or PFPT. We observed moderate correlations between the concentrations of serum IgG measured by RID and those measured in colostrum by RID (*r*_s_ 0.542; *P* = .01) and DR (*r*_s_ 0.512; *P* = .01) and OR (*r*_s_ 0.503; *P* = .01). However, our main goal was to define optimal cut‐off points for the identification of poor‐quality colostrum. These were ≤23.75% and 23.90% for the DR and OR, respectively, ie, higher by about 1% than the cut‐off previously proposed for the OR.^12^ Although the variation is minimal, the use of an optimal cut‐point for colostral quality should increase the percentage of foals acquiring adequate passive immunity.

## CONFLICT OF INTERESTS

No competing interests have been declared.

## AUTHOR CONTRIBUTIONS

E. Rampacci contributed to conceptualisation, methodology, investigation, formal analysis, writing of the original draft manuscript and visualisation. K. Mazzola contributed to conceptualisation, methodology, investigation, writing–review and editing. F. Beccati contributed to formal analysis, validation, visualisation, writing–review and editing. F. Passamonti contributed to conceptualisation, methodology, supervision, validation, data integrity, funding acquisition, project administration, writing–review and editing. All authors had full access to all study data and they have given final approval to the manuscript.

## INFORMED CONSENT

Owners gave their informed consent for their horses to be included in the study.

## ETHICAL ANIMAL RESEARCH

Research ethics committee oversight not currently required by this journal: The study was performed on material collected during clinical procedures.

### PEER REVIEW

The peer review history for this article is available at https://publons.com/publon/10.1111/evj.13568.

## Supporting information

Table S1

## Data Availability

The data that support the findings of this study are available on request from the corresponding author.

## References

[1] Jeffcott LB . Some practical aspects of the transfer of passive immunity to newborn foals. Equine Vet J. 1974;6:109–15. 4137197 10.1111/j.2042-3306.1974.tb03942.x

[2] Tyler‐McGowan CM , Hodgson JL , Hodgson DR . Failure of passive transfer in foals: incidence and outcome on four studs in New South Wales. Aust Vet J. 1997;75:56–9. 9034501 10.1111/j.1751-0813.1997.tb13832.x

[3] Perkins GA , Wagner B . The development of equine immunity: current knowledge on immunology in the young horse. Equine Vet J. 2015;47:267–74. 25405920 10.1111/evj.12387

[4] Giguère S , Polkes AC . Immunologic disorders in neonatal foals. Vet Clin North Am Equine Pract. 2005;21:241–72. 16051049 10.1016/j.cveq.2005.04.004

[5] Liepman RS , Dembek KA , Slovis NM , Reed SM , Toribio RE . Validation of IgG cut‐off values and their association with survival in neonatal foals. Equine Vet J. 2015;47:526–30. 25683641 10.1111/evj.12428

[6] Ujvari S , Schwarzwald CC , Fouché N , Howard J , Schoster A . Validation of a point‐of‐care quantitative equine IgG turbidimetric immunoassay and comparison of IgG concentrations measured with radial immunodiffusion and a point‐of‐care IgG ELISA. J Vet Intern Med. 2017;31:1170–7. 28561898 10.1111/jvim.14770 PMC5508326

[7] Jeffcott LB , Jeffcott TJ . Studies on passive immunity in the foal. III. The characterization and significance of neonatal proteinuria. J Comp Pathol. 1974;84:455–65. 4142878 10.1016/0021-9975(74)90038-3

[8] Morris DD , Meirs DA , Merryman GS . Passive transfer failure in horses: incidence and causative factors on a breeding farm. Am J Vet Res. 1985;46:2294–9. 4073639

[9] Kohn CW , Knight D , Hueston W , Jacobs R , Reed SM . Colostral and serum IgG, IgA, and IgM concentrations in Standardbred mares and their foals at parturition. J Am Vet Med Assoc. 1989;195:64–8. 2759897

[10] Kummer LL , Govaere J , Egri B . Comparison of the reliability of snap foal Ig test, Gamma‐Check E test, refractometry and electrophoresis for determining the immune status of newborn foals in the first hours of life. Acta Vet Hung. 2018;66:573–86. 30580538 10.1556/004.2018.051

[11] Cash RSG . Colostral quality determined by refractometry. Equine Vet Educ. 1999;11:36–8.

[12] Chavatte P , Clement F , Cash R , Grongnet JF . Field determination of colostrum quality by using a novel, practical method. Proc Am Assoc Equine Pract. 1998;44:206–9.

[13] Elsohaby I , Riley CB , McClure JT . Usefulness of digital and optical refractometers for the diagnosis of failure of transfer of passive immunity in neonatal foals. Equine Vet J. 2019;51:451–7. 30417417 10.1111/evj.13040

[14] Hajian‐Tilaki K . Sample size estimation in diagnostic test studies of biomedical informatics. J Biomed Inform. 2014;48:193–204. 24582925 10.1016/j.jbi.2014.02.013

[15] Hess AS , Shardell M , Johnson JK , Thom KA , Strassle P , Netzer G , et al. Methods and recommendations for evaluating and reporting a new diagnostic test. Eur J Clin Microbiol Infect Dis. 2012;31:2111–6. 22476385 10.1007/s10096-012-1602-1 PMC3661219

[16] Hinkle D , Wiersma W , Jurs S . Applied Statistics for the Behavioral Sciences, 5th edn. Boston: Houghton Mifflin; 2003.

[17] Mukaka MM . Statistics corner: a guide to appropriate use of correlation coefficient in medical research. Malawi Med J. 2012;24:69–71. 23638278 PMC3576830

[18] Erhard MH , Luft C , Remler HP , Stangassinger M . Assessment of colostral transfer and systemic availability of immunoglobulin G in new‐born foals using a newly developed enzyme‐linked immunosorbent assay (ELISA) system. J Anim Physiol Anim Nutr (Berl). 2001;85:164–73. 11686785 10.1046/j.1439-0396.2001.00313.x

[19] Rampacci E , Passamonti F , Bottinelli M , Stefanetti V , Cercone M , Nannarone S , et al. Umbilical infections in foals: microbiological investigation and management. Vet Rec. 2017;180:543. 28314783 10.1136/vr.103999

[20] Pisello L , Forte C , D’Avino N , Pisano R , Hyatt DR , Rueca F , et al. Evaluation of Brix refractometer as an on‐farm tool for colostral IgG evaluation in Italian beef and dairy cattle. J Dairy Res. 2021;88:189–93. 33952363 10.1017/S0022029921000315

[21] Buderer NM . Statistical methodology: I. Incorporating the prevalence of disease into the sample size calculation for sensitivity and specificity. Acad Emerg Med. 1996;3:895–900. 8870764 10.1111/j.1553-2712.1996.tb03538.x

[22] Bujang MA , Adnan TH . Requirements for minimum sample size for sensitivity and specificity analysis. J Clin Diagn Res. 2016;10:YE01–6. 10.7860/JCDR/2016/18129.8744 PMC5121784 27891446

