# Supplementary material for: Diagnostic characteristics of refractometry cut‐off points for the estimation of immunoglobulin G concentration in mare colostrum
Source: Equine Vet J. 2022 Mar 8;55(1):102–10. doi: 10.1111/evj.13568 (PMC10078710; doi:10.1111/evj.13568)
Supplement: Supplementary file 1 — Table S1 [file EVJ-55-102-s001.pdf]

Table S1. IgG concentrations measured by radial immunodiffusion in colostrum and sera and values read by the optical and digital refractometers.

Animals	DR results for colostrum	OR results for colostrum	Colostral IgG measured by RID	DR results for serum	OR results for serum	Serum IgG measured by RID
Foal 1	27.2	27.6	9118.466363	10	9.8	1692.35034
Foal 2	16.8	16.6	1817.615651	8.1	8	574.0629013
Foal 3	25.8	25.2	7211.275569	8.6	8.6	934.3264776
Foal 4	27.3	27.2	7822.171353	10.9	10.8	2812.291871
Foal 5	23.7	23.8	4067.424958	7.8	7.6	301.1080462
Foal 6	28.6	27.8	8950.996944	9.3	9.2	1878.404788
Foal 7	28	28.2	7211.275569	9.7	9.6	1692.35034
Foal 8	21.5	21.2	5141.598471	8.1	7.8	454.4039127
Foal 9	20.2	20.2	3218.150599	19.9	19.9	617.053541
Foal 10	35.3	>32	11249.16748	9.9	9.8	1254.00931
Foal 11	40.4	>32	14650.26696	9	8.8	1692.35034
Foal 12	13.6	13	716.9732767	6.6	6.4	72.98180538
Foal 13	36.5	>32	8950.996944	9.5	9.2	1692.35034
Foal 14	36.7	>32	16860.45463	8	7.9	1192.39281
Foal 15	20.5	20.6	3421.156259	8.4	8.2	934.3264776
Foal 16	26.7	26.2	10155.87813	8	8	1073.816274
Foal 17	30.3	30.2	11249.16748	9.7	9.8	2719.63227
Foal 18	23.6	23.2	7822.171353	7.6	7.4	617.053541
Foal 19	25.3	25.4	8457.901617	7.5	7.2	1045.142238
Foal 20	25.7	25.4	9630.18755	7.6	7.4	1223.00704
Foal 21	33.5	>32	13809.65222	10.8	10.6	2114.475404
Foal 22	36.8	>32	12595.29478	10.1	9.8	2074.160205
Foal 23	27.1	27	10155.87813	9.5	9.2	1955.542838
Foal 24	24	23.8	7211.275569	8.9	8.8	1802.818892
Foal 25	21.3	20.8	6063.987442	8.4	8.2	417.6218933
Foal 26	27.2	26	8620.714571	9.3	9.2	1448.17174
Foal 27	31.2	30.8	12009.07015	9.9	9.6	3298.872201
Foal 28	27.7	27.4	10695.53811	9.8	9.6	1656.303566
Foal 29	33.9	>32	14226.85528	9.4	9.2	2114.475404
Foal 30	30.4	30	13195.4888	11.2	11	2628.524824
Foal 31	26	25.8	9803.865589	8	8	661.5963356

Foal 32	22.7	22.8	6482.579327	8.7	8.8	829.7193379
Foal 33	24.5	23.8	9979.095783	10	9.8	1916.779794
Foal 34	29	28.8	9458.061666	8.2	8	988.9582801
Foal 35	27.2	27	10878.52908	8.7	9	1045.142238
Foal 36	27.2	27	8785.07968	7.2	7	301.1080462
Foal 37	31.3	30.4	11249.16748	9.5	9.2	2074.160205
Foal 38	29.8	29.4	10334.21264	8.1	8	1381.898775
Foal 39	23.6	23.4	7211.275569	9	8.8	1550.491479
Foal 40	24.8	24.4	7822.171353	8.2	8.2	1481.890281
Foal 41	23.9	23	7211.275569	8.7	8.6	2279.616591
Foal 42	22.9	22.4	4892.028161	8.1	8	454.4039127
Foal 43	29	28.8	10878.52908	9	8.8	1192.39281
Foal 44	20.7	20.8	5016.037239	9.4	9.2	1073.816274
Foal 45	24	24	15515.71617	10	9.8	1162.166618
Foal 46	20.6	20	3218.150599	7.5	7.4	229.5250324
Foal 47	31.3	31	13809.65222	10.4	10.2	2398.90955
Foal 48	23	23.2	5792.686961	9.1	9	1692.35034
Foal 49	14.7	14.8	1817.615651	8.6	8.4	804.5376499
Foal 50	28.2	28	11249.16748	9.8	9.6	2114.475404
Foal 51	27.8	27.8	9287.487937	10.9	10.8	1840.41782
Foal 52	42.9	>32	24698.43097	11.6	11.4	3148.823695
Foal 53	19.8	20	5397.377402	9.1	9.2	1254.00931
Foal 54	23.8	23.8	7667.119174	10.2	10.2	1916.779794
Foal 55	30.5	30.5	14226.85528	10.6	10.4	2812.291871
Foal 56	22.9	23.2	6482.579327	9.5	9.2	2034.233044
Foal 57	25	25.4	9803.865589	8.9	8.8	1381.898775
Foal 58	45	>32	23603.26281	10	9.8	1955.542838
Foal 59	26.4	26.2	8785.07968	9.3	9.2	1728.785152
Foal 60	33.1	>32	13195.4888	9.8	9.6	1317.177965
Foal 61	31.8	31.6	14226.85528	9.8	9.6	2538.969533
Foal 62	28.2	28.2	8620.714571	9.4	9.4	2321.871984
Foal 63	32.6	>32	12595.29478	10.4	10.6	684.4497911
Foal 64	29	29	9979.095783	11	10.8	2407.546887
Foal 65	27.9	27.8	10514.0993	10.3	10	2494.773946

Foal 66	35.7	>32	11436.81492	8.4	8.2	1102.87835
Foal 67	22.3	22.4	5016.037239	8.9	8.9	1448.17174
Foal 68	27.6	27.6	500.4881987	8.8	8.8	707.6912853
Foal 69	22.3	22.2	8950.996944	10.6	10.4	1955.542838
Foal 70	34.1	>32	7513.61915	10.3	10	1765.608003
Foal 71	22.7	22.6	5141.598471	8.2	8	617.053541
Foal 72	29.5	29.2	12398.33442	9.3	9.4	1414.841238
Foal 73	32.8	>32	12595.29478	11.2	11.2	2279.616591
Foal 74	21.7	21.4	6063.987442	8.6	8.4	1515.996861
Foal 75	31.5	31.4	11816.76625	10.3	10	1620.644832
Foal 76	38.9	>32	22269.2261	10.3	10.2	2494.773946
Foal 77	29.4	30.2	8296.640819	10.4	10.2	1448.17174
Foal 78	35.5	>32	15297.02563	9.4	9.4	1955.542838
Foal 79	21.8	21.8	6625.214265	10.1	9.8	2114.475404
Foal 80	31.8	32	14017.47767	10.1	9.8	1802.818892
Foal 81	33	>32	12202.92621	9.1	9	1802.818892
**Abbreviations**: DR, digital refractometer; OR, optical refractometer; RID, radial immunodiffusion.						
